# Acute Severe Hypoxia Decreases Mitochondrial Chain Complex II Respiration in Human Peripheral Blood Mononuclear Cells

**DOI:** 10.3390/ijms26020705

**Published:** 2025-01-15

**Authors:** Marianne Riou, Anne-Laure Charles, Irina Enache, Charles Evrard, Cristina Pistea, Margherita Giannini, Anne Charloux, Bernard Geny

**Affiliations:** 1 Biomedicine Research Center of Strasbourg (CRBS), UR 3072, “Mitochondria, Oxidative Stress and Muscle Plasticity”, Faculty of Medicine, University of Strasbourg, 67000 Strasbourg, France; marianne.riou@chru-strasbourg.fr (M.R.); anne.laure.charles@unistra.fr (A.-L.C.); irina.enache@chru-strasbourg.fr (I.E.); evrard.charles@outlook.com (C.E.); cristina.pistea@yahoo.fr (C.P.); margherita.giannini@chru-strasbourg.fr (M.G.); anne.charloux@chru-strasbourg.fr (A.C.); 2 Department of Physiology and Functional Explorations, University Hospital of Strasbourg, 67091 Strasbourg, France

**Keywords:** hypoxia, PBMCs, mitochondrial respiration, reactive oxygen species, complex II, inflammation, succinate dehydrogenase

## Abstract

Peripheral blood mononuclear cells’ (PBMCs) mitochondrial respiration is impaired and likely involved in myocardial injury and heart failure pathophysiology, but its response to acute and severe hypoxia, often associated with such diseases, is largely unknown in humans. We therefore determined the effects of acute hypoxia on PBMC mitochondrial respiration and ROS production in healthy volunteers exposed to controlled oxygen reduction, achieving an inspired oxygen fraction of 10.5%. We also investigated potential relationships with gene expression of key biomarkers of hypoxia, succinate and inflammation, as hypoxia and inflammation share common mechanisms involved in cardiovascular disease. Unlike global mitochondrial respiration, hypoxemia with a spO_2_ ≤ 80% significantly reduced PBMC complex II respiration (from 6.5 ± 1.2 to 3.1 ± 0.5 pmol/s/10^6^ cell, *p* = 0.04). Complex II activity correlated positively with spO_2_ (r = 0.63, *p* = 0.02) and inversely correlated with the succinate receptor SUCNR1 (r = −0.68), the alpha-subunit of the hypoxia-inducible factor (HIF-1α, r = −0.61), the chemokine ligand-9 (r = −0.68) and interferon-stimulated gene 15 (r = −0.75). In conclusion, severe hypoxia specifically impairs complex II respiration in association with succinate, inflammation and HIF-1α pathway interactions in human PBMCs. These results support further studies investigating whether modulation of complex II activity might modify the inflammatory and metabolic alterations observed in heart failure.

## 1. Introduction

Efficient cellular oxygen delivery is essential for the survival of most animals and humans, and hypoxia is implicated in many pathologies ranging from pulmonary and cardiovascular diseases to cancer. Hypoxia can also occur, or is even provoked, in settings implying healthy volunteers, such as high mountain trekking and/or hypoxic training, aiming to improve blood oxygen transport [1,2].

Three major acute or chronic responses occur in response to hypoxia. First, hypoxic pulmonary vasoconstriction (HPV) can be beneficial and optimize pulmonary gas exchange and blood oxygenation when alveolar hypoxia is localized and transient, but HPV is also potentially detrimental when the hypoxia is prolonged and generalized [3]. Secondly, acute hyperventilation induced by carotid body chemoreceptors should enable one to maintain tissue oxygenation as far as possible. Finally, at the cellular level, cells adapt their metabolism to survive. Interestingly, cellular adaptations are not only limited to tissues.

Circulating cells, largely involved in oxygen transport, immunity, inflammation, and systemic coordination, also adapt their function to hypoxia. Particularly, immune cells such as peripheral blood mononuclear cells (PBMCs), i.e., lymphocytes, monocytes and dendritic cells, undergo rapid metabolic adaptation to maintain immune homeostasis in hypoxic environments. Furthermore, the metabolic state of immune cells can determine their immune function [4]. In addition to their easy accessibility through a small blood sample, PBMCs have emerged as potential biomarkers in several pathologies [5,6,7], including acute conditions such as heart failure or septic shock [8,9].

Particularly, there is ongoing work investigating the potential implication of PBMCs’ impairment in myocardial and more generally in cardiovascular disease, and recent data suggest that PBMC’s mitochondrial respiration is involved in the pathophysiology of this major public health issue [8,10,11,12]. Interestingly, hypoxia can be one of the characteristics of severe myocardial and cardiovascular disease, leading to dyspnea, corner symptoms in many patients. However, PBMCs’ mitochondrial respiration response to acute and severe hypoxia is largely unknown in humans. Thus, investigating PBMCs’ mitochondrial respiration is an interesting approach to better characterize diseases and potentially open up new therapeutic avenues, such as in cardiorespiratory diseases [10,11,13,14]. For example, reduced mitochondrial respiratory chain complex II (succinate dehydrogenase [SDH]) activity might be associated with subclinical diastolic dysfunction after heart transplantation and thus related to heart failure pathophysiology [15].

Indeed, because they consume the majority of cellular oxygen (O_2_), mitochondria are considered ideal candidates to be the first acute O_2_ detectors in specialized cells, including in immune cells where they play a role in the regulation and modulation of immune responses [16,17]. Moreover, mitochondria are crucial to producing energy. They are the site of the electron transport chain (ETC), which generates adenosine triphosphate (ATP) through oxidative phosphorylation (OXPHOS) via the activity of five complexes. This process consumes oxygen in proportion to the rate of ATP utilization by cells. In normoxia, performance of the ETC usually depends mainly on oxidation of nicotinamide adenine dinucleotide (NAD)-related substrates that supply the mitochondrial complex I to initiate OXPHOS. Mitochondria also release reactive oxygen species (ROS), including the non-radical hydrogen peroxide (H_2_O_2_), which act as signaling molecules in a variety of functional responses [3,18].

Under hypoxia, cells can switch their metabolism towards glycolysis, but the effect of hypoxia on mitochondrial respiratory chain respiration and ROS production is largely unknown. Interestingly, the molecular signaling pathways that modulate the response to hypoxia and inflammation share significant crosstalk, highlighting the rationale for studying both parameters during hypoxia [19,20]. Accordingly, ETC complex II has been suggested to be involved in responses to acute hypoxia but also to inflammation in various cell types, as it metabolizes succinate, which may stabilize the alpha-subunit of the hypoxia-inducible factor (HIF-1α), a transcription factor that regulates genes involved in glucose uptake and metabolism, cell survival, vascular tone, apoptosis or cell adhesion [21,22,23]. A better understanding of these effects is warranted as cellular hypoxia or pseudohypoxia is one of the mechanisms underlying many chronic diseases such as cancer or pulmonary arterial hypertension (PAH) [24,25,26].

The aim of this study was therefore to investigate, for the first time, the effects of acute and severe hypoxia on mitochondrial respiration and mitochondrial ROS production in human PBMCs. Particularly, we investigated whether ETC complex II activity might be impaired, in relation to the succinate pathway, HIF-1α and biomarkers of inflammation such as the signal transducers and activators of transcription-3 (STAT3) and interferon genes such as chemokine ligand-9 (CXCL9) and interferon-stimulated gene 15 (ISG15). Indeed, all these pathways might be involved in cardiovascular disease pathology [27,28,29].

## 2. Results

### 2.1. Subjects’ Baseline Clinical Characteristics and Cardiorespiratory Effects of Hypoxia

Thirteen healthy male volunteers, with a mean age 29.5 ± 1.7 and mean body mass index 24.8 ± 1 kg/m^2^, were prospectively enrolled in the study. At baseline, mean oxygen saturation measured by non-invasive pulse oximetry (spO_2_) was 99 ± 1%, mean heart rate was 73 ± 2/min and systemic blood pressure was 135/75 ± 3 mmHg.

Decreasing the inspired oxygen fraction (FiO_2_) resulted in significant changes in spO_2_ and heart rate (*p* < 0.001 and *p* = 0.04, respectively). At FiO_2_ 10.5%, mean spO_2_ was 82.9 ± 1.8%, and five subjects had spO_2_ ≤ 80%, defined as severe acute hypoxemia [30]. Mean respiratory rate was 11 ± 1/min and mean minute ventilation was 11.5 ± 1 L/min, with no significant difference between subjects with spO_2_ ≤ 80% and the others, although there was a tendency for a lower minute ventilation in subjects with spO_2_ ≤ 80% (*p* = 0.66 and *p* = 0.06, respectively). However, no significant ventilatory adaptation was observed between t-start and t-hypoxia regarding the individual values of the subjects and regardless of the degree of hypoxemia: respiratory rate variation of −2.5 ± 0.6/min and ventilation variation of +1.15 ± 0.9 L/min in the total population.

The clinical parameters are summarized in Table 1.

### 2.2. Severe Hypoxia Significantly and Specifically Reduced Complex II of the Mitochondrial Respiratory Chain Respiration

**Figure 1:**
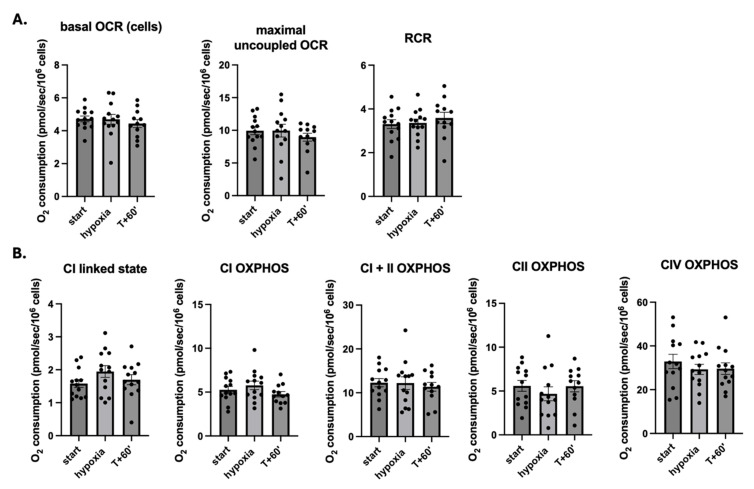
Basal, maximal, coupling (RCR) (**A**) and complex-linked mitochondrial respiration (**B**) responses to hypoxia in the entire population at t-start, hypoxia and t + 60′. All the results are expressed as mean ± SEM. OCR: oxygen consumption ratio; OXPHOS: oxidation phosphorylation; RCR: respiratory control ratio.

Nevertheless, when considering the whole population, no significant difference was observed in terms of basal or maximal respiration or respiratory control ratio (RCR) (Figure 1A) and in the activity of the ETC complexes in PBMCs after hypoxia, especially in the activity of complexes I and IV. Nevertheless, complex II tended to decrease in t-hypoxia with a greater dispersion between values, although not reaching statistical significance, as shown in Figure 1B.

To go further, we analyzed the mitochondrial ETC complexes’ respiration in the five subjects presenting with a spO_2_ ≤ 80% during hypoxia and compared them with the subjects without a significant drop in their spO_2_. No difference in the activity of ETC complexes I, I + II and IV was observed between these five subjects and the eight subjects with spO_2_ > 80% during t-hypoxia.

**Figure 2:**
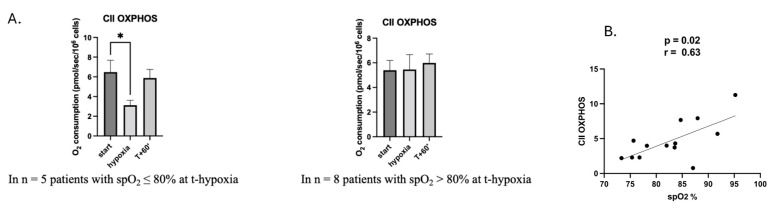
Complex II mitochondrial respiration is impaired when spO_2_ decreases under 80%. (**A**) Mitochondrial complex II activity (CII OXPHOS) in PBMCs from the healthy volunteers with spO_2_ ≤ 80% or spO_2_ > 80% at t-start, hypoxia and t + 60′. (**B**) Positive correlation between the ETC complex II respiration (CII OXPHOS) and the spO_2_ values. All the results are expressed as mean ± SEM. * *p* = 0.04. OXPHOS: oxidation phosphorylation; spO_2_: oxygen saturation measured by non-invasive pulse oximetry.

Interestingly however, considering complex II activity, we observed a significant decrease in the response to acute hypoxemia when spO_2_ was ≤80%. In these subjects, complex II respiration of PBMCs decreased by 52% (from 6.5 ± 1.2 at t-start to 3.1 ± 0.5 pmol/s/10^6^ cell at t-hypoxia) and returned to baseline at the end of the study, *p* = 0.04, Figure 2A. Additionally, complex II respiration correlated positively with spO_2_ values (r = 0.63, *p* = 0.02); Figure 2B. In contrast, complex II activity was not modified under hypoxia in the eight subjects with spO_2_ > 80%: mean O_2_ consumption (in pmol/s/10^6^ cell) of 5.4 ± 0.8 at t-start, 5.5 ± 1.2 at t-hypoxia and 5.9 ± 0.7 at t + 60′; *p* = 0.96, Figure 2A.

### 2.3. H_2_O_2_ Production Was Not Modified by Hypoxia

**Figure 3:**
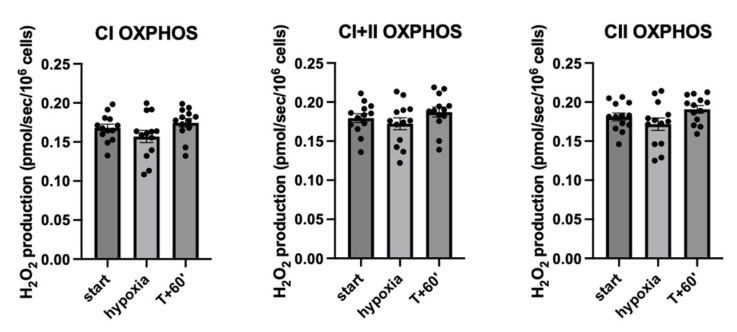
Hypoxia did not modify mitochondrial production of hydrogen peroxide (H_2_O_2_). H_2_O_2_ produced by mitochondrial respiration in PBMCs from the 13 healthy volunteers at t-start, hypoxia and t + 60′ in CI, CI+II and CII OXPHOS. All the results are expressed as mean ± SEM. OXPHOS: oxidation phosphorylation.

Regarding H_2_O_2_ production in PBMCs during mitochondrial respiration, no difference was observed between t-start, t-hypoxia and t + 60′: mean H_2_O_2_ production (in pmol/s/10^6^ cell) in complex I OXPHOS of 0.17 ± 0.004 at t-start, 0.16 ± 0.07 at t-hypoxia and 0.17 ± 0.05 at t + 60′, *p* = 0.11 and mean H_2_O_2_ production in complex II OXPHOS of 0.18 ± 0.004 at t-start, 0.17 ± 0.007 at t-hypoxia and 0.19 ± 0.004 at t + 60′, *p* = 0.12, regardless of the degree of desaturation of the subjects, as shown in Figure 3.

### 2.4. OXPHOS Complex II Activity in Hypoxia Is Inversely Correlated to SUCNR1, HIF-1α, ISG15 and CXCL9 Gene Expression in PBMCs

To complete our analysis, knowing that acute hypoxia and inflammation share common cellular response mechanisms [19,20], we examined the expression of genes encoding for HIF-1α and genes involved in inflammation: signal transducer and activator of transcription 3 (STAT3) and interferon type 1 and 2 genes: chemokine ligand-9 (CXCL9) and interferon-stimulated gene 15 (ISG15). We also determined the gene expression of the succinate receptor 1, SUCNR1, because it is activated by intracellular succinate levels [31]. Analyses were performed in PBMCs from eight healthy subjects (n = 4 with spO_2_ > 80% and n = 4 with spO_2_ ≤ 80%).

**Figure 4:**
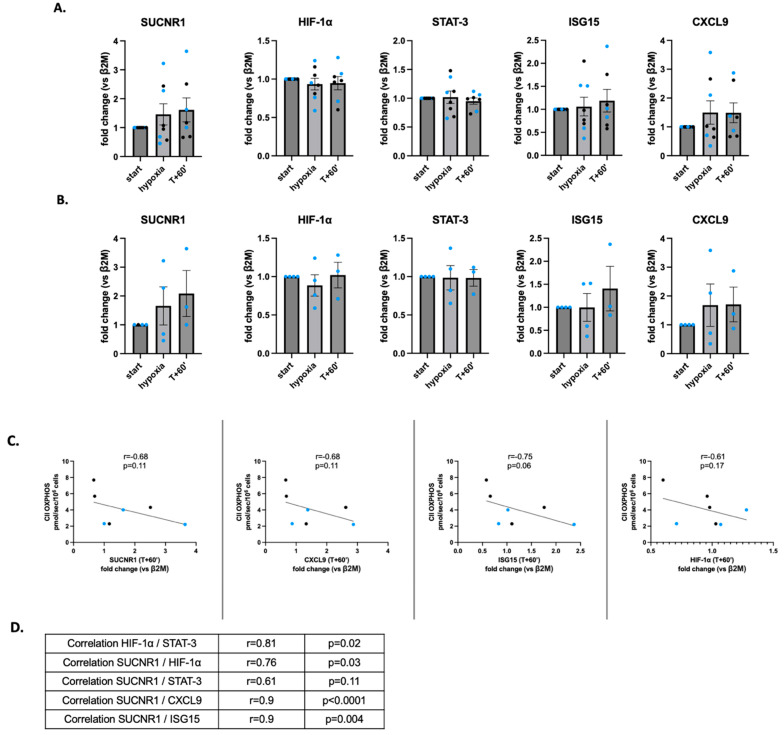
Gene expression variations in SUCNR1, HIF-1α, ISG15, CXCL9 and STAT3 in PBMCs and correlation with OXPHOS complex II. (**A**) Comparison of gene expression between t-start, hypoxia and t + 60′ in 8 subjects. (**B**) Comparison of gene expression between t-start, hypoxia and t + 60′ in the 4 subjects with spO_2_ ≤ 80%. Analysis at t + 60′ is missing in one subject. (**C**) Complex II OXPHOS at t-hypoxia is inversely correlated with SUCNR1, HIF-1α, CXCL9 and ISG15 expression at t + 60′. (**D**) Correlations between the expressions of HIF-1α and STAT3 and SUCNR1 and the 4 other analyzed genes. Blue dots represent values for subjects with spO_2_ ≤ 80% and dark dots represent values for subjects with spO_2_ > 80%. β2M: beta-2-microglobulin; CXCL9: chemokine ligand-9; HIF-1α: alpha-subunit of the hypoxia-inducible factor; ISG15: interferon-stimulated gene 15; STAT3: signal transducer and activator of transcription 3; SUCNR1: ligand–receptor pair succinate receptor 1.

First, when analyzing the variation in the expression of the five genes at t-start, t-hypoxia and t + 60′, we found no significant changes between the three times (Figure 4A), including in the four subjects analyzed with spO_2_ ≤ 80% (Figure 4B).

We further investigated a link between complex II OXPHOS activity and the cellular state induced by hypoxia, particularly in subjects with spO_2_ ≤ 80%. Indeed, complex II sits at the interface between the ETC and the tricarboxylic acid cycle and metabolizes succinate, which is a critical signal for modulating the inflammatory process [32]. Therefore, we analyzed the gene expression of SUCNR1, as a reflection of intracellular succinate variation. We found no significant correlation between SUCNR1 gene expression and complex II OXPHOS at t-hypoxia (*p* = 0.97), but considering the time of cell adaptation to hypoxia, the value of complex II OXPHOS in hypoxia was negatively correlated with SUCNR1 (r = −0.68), HIF-1α (r = −0.61), CXCL9 (r = −0.68) and ISG15 (r = −0.75) gene expression at t + 60′ (Figure 4C).

Interestingly, SUCNR1 was significantly positively correlated with the inflammatory state of PBMCs, as reflected by STAT3, CXCL9 and ISG15, and correlated with HIF-1α (Figure 4D). Complex II and STAT3 were less correlated (r = −0.21). In any event, these data strengthen the link between succinate–inflammation and HIF-1α in PBMCs, modulated by complex II activity.

## 3. Discussion

The main result of this study is that acute transient hypoxemia with a spO_2_ ≤ 80% significantly reduced complex II activity in human PBMCs, while the activity of other ETC complexes remained unchanged. Furthermore, complex II activity was positively correlated with spO_2_ levels and inversely correlated with the gene expression of SUCNR1, HIF-1α, CXCL9 and ISG15, strengthening the link between the complex II–succinate pathway and inflammation and HIF-1α in PBMCs during hypoxia.

### 3.1. Cardiorespiratory Responses to Hypoxia

Oxygen saturation measured by non-invasive pulse oximetry is related to the arterial oxygen partial pressure (pO_2_) by the sigmoidal oxygen–hemoglobin dissociation curve. Thus, a spO_2_ ≤ 80% corresponds to deep hypoxemia, i.e., a pO_2_ < 50 mmHg, and pO_2_ decreases rapidly with decreasing oxygen saturation [30]. In our protocol, 38% of subjects achieved spO_2_ ≤ 80% during hypoxia. This result was not unexpected, as inter-individual differences in ventilatory adaptation between subjects result in variable responses to hypoxia. Interestingly, although not significant, subjects with spO_2_ ≤80% did not have an increase in minute ventilation compared to the others, which may have contributed to the greater desaturation observed in these patients. In addition, the subjects stayed comfortably resting in a room at constant temperature during the protocol, which probably contributed to the lack of significant variation (increase) in respiratory rate and minute ventilation, as described by Hermand et al. [33].

### 3.2. Effects of Hypoxia on PBMCs’ Mitochondrial Respiration

#### 3.2.1. Global Mitochondrial Respiration and ETC Complex Respiration

In our study, we did not observe any change in global mitochondrial respiration in PBMCs, including basal and maximal respiratory capacity, mitochondrial coupling and ETC complex I, III and IV activities. To date, mitochondrial respiration in human PBMCs during acute hypoxia has not been specifically investigated in previous studies. Studies on pulmonary vascular cells have suggested a role for mitochondrial complex IV in oxygen sensing and signal transduction of HPV, with hypoxia inhibiting electron transfer from complex IV to oxygen [34]. In addition, adaptation to hypoxic stress in the brain cortex would induce a switch from complex I to complex II OXPHOS [23]. However, the short duration of hypoxia in our protocol may explain the lack of change in complex I or IV activity.

#### 3.2.2. Mitochondrial Complex II Respiration and Link to Inflammation and HIF-1α

Interestingly, acute and severe hypoxemia (spO_2_ ≤ 80%) was associated with a significant reduction in complex II activity in PBMCs. Although this is the first report in healthy humans, it appears to be consistent with a publication reporting similar results during exacerbation of chronic obstructive pulmonary disease [35]. Accordingly, Sharma et al. showed that inhibition of mitochondrial complex II induces hypoxic gene expression [36]. Complex II sits at the interface between the ETC and the tricarboxylic acid cycle and metabolizes succinate to fumarate. It requires oxidized flavin adenine dinucleotide (FAD) and NAD^+^ as cofactors. However, when these are predominantly present in their reduced form, as in hypoxia, the function of complex II is impaired [37].

### 3.3. Decreased PBMCs’ CII Respiration Is Associated with Increased Succinate Receptor 1, HIF-1α Activation and Markers of Inflammation

**Figure 5:**
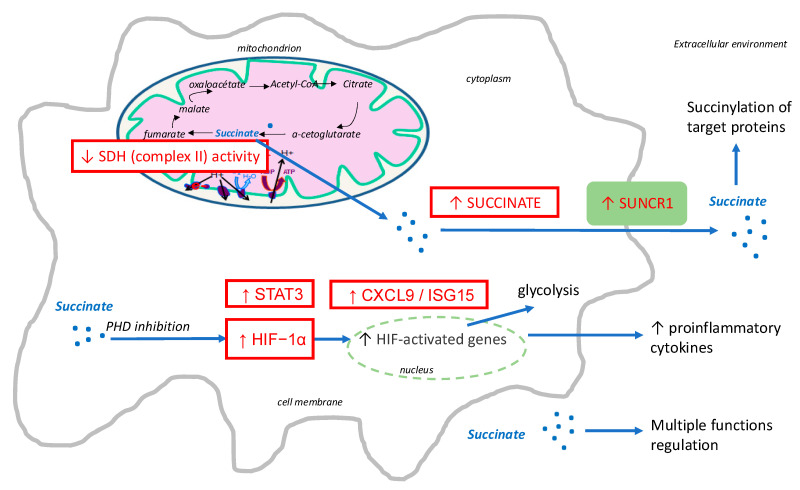
Schematic representation of the complex II (succinate dehydrogenase [SDH])–succinate–hypoxia inducible factor (HIF) and ligand–receptor pair succinate receptor 1 (SUCNR1) axis involved in the response to acute hypoxia in PBMCs, extrapolated from our data and the literature. Acute hypoxia induces a decrease in SDH activity, leading to intracellular succinate accumulation and SUCNR1 expression and activation, in synergy with several inflammatory signaling cascades reflected by increased signal transducer and activator of transcription 3 (STAT3), chemokine ligand-9 (CXCL9) and interferon-stimulated gene 15 (ISG15) gene expression. Succinate is transported into the cytoplasm, where it inhibits prolyl hydroxylase domain (PHD) enzyme function, resulting in stabilization of HIF-1α and increasing the expression of genes that have HIF-response elements such as glycolysis genes. Further succinate is exported into the local extracellular environment, where it accumulates and binds and activates SUCNR1, whose signal is in synergy with several inflammatory signaling cascades.

According to the literature, one of the consequences of decreased CII activity is the intracellular abnormal accumulation of succinate, which acts as an oncometabolite involved in tumorigenesis and cancer progression, and which is a critical signal for modulating the inflammatory process [38]. In our study, we confirmed the link between CII OXPHOS activity and succinate by analyzing SUCNR1 and showed that a decrease in CII OXPHOS was associated with higher gene expression of SUCNR1 in PBMCs, suggesting an increase in intracellular succinate. Since succinate accumulation can stabilize the critical transcription factor of the cellular response to hypoxia, HIF-1α, via the SUCNR1 [39], as shown in activated macrophages [32], we examined the gene expression of HIF-1α which was inversely correlated with CII OXPHOS, supporting the link between CII reduction, succinate accumulation and HIF-1α activation. Interestingly, in our study, SUCNR1 was not only strongly positively correlated with HIF-1α gene expression but also with the expression of STAT3, CXCL9 and ISG15, three genes involved in inflammatory pathways. These results are consistent with the previous literature, as HIFs have been shown to be key elements in the control of immune cell metabolism and function. Kammerer et al. confirmed the critical role of immune response to acute hypoxia in the symptoms of high-altitude pathologies, such as acute mountain sickness [40]. In macrophages, HIF-1α increases cell motility and the expression of pro-inflammatory cytokines such as interleukin 1β or tumor necrosis factor-α [41,42]. In addition, previous publications reported a crosstalk between Janus kinase (JAK)/STAT3 and hypoxia/HIF-1α pathways [43,44,45], and some studies have shown that STAT3 regulates HIF-1α transcriptional activity [46]. In our study, we also found a strong positive correlation between STAT3 and HIF-1α, and CII OXPHOS was inversely correlated with CXCL9 and ISG15, supporting the link between reduced CII activity and inflammation. The mechanisms linking CII, succinate, HIF-1α and inflammation are summarized in Figure 5.

In terms of clinical relevance, complex II impairment has been shown to be associated with diseases including cancer or heart disease [15,38,47,48]. Interestingly, PBMCs represent several immune populations, which have different roles in inflammation and may respond differently to hypoxia, suggesting a potential specific response of monocytes or lymphocytes [49,50]. Another type of circulating cell, platelets from patients with PAH (group 1 pulmonary hypertension [PH]), have increased expression and activity of complex II, the role of which in the pathophysiology remains to be explored [51]. Further studies are therefore warranted to investigate whether the decrease in complex II is involved in common pathophysiological mechanisms, as a potential link to HIF stabilization through succinate accumulation in hypoxia, but also in some cases in normoxia. Indeed, some pathologies induce a pseudohypoxic state of the cells as cancer or during the “normobaric oxygen paradox” defined by a “hypoxia-like” response to the return to normoxia after a hyperoxic event, which is characterized by the activation of numerous pathways in PBMCs, including HIF-1 [52,53].

### 3.4. Effects of Hypoxia on H_2_O_2_ Production by PBMCs

As redox reactions are involved in cell signaling pathways through ROS generation, we also investigated the levels of H_2_O_2_ produced by mitochondrial respiration in PBMCs, and we observed no change. These results are consistent with our findings on the activity of ETC complexes, where the activity of complexes I and III, main mitochondrial sources of ROS, was not modified by hypoxia. However, other markers of the pro-antioxidant balance might be modified.

### 3.5. Limitations

Despite our interesting findings, this study has some limitations. Firstly, our analyses were limited to 13 healthy male participants, with no inclusion of women. As we know that sex differences may influence physiological responses to various stressors through the influence of sex hormones [54], future research should explore potential sex-related differences in the response of PBMCs to hypoxia. Indeed, to the best of our knowledge, there are no human data investigating the sex-specific response of PBMC mitochondrial respiration to hypoxia. However, in rats, chronic intermittent hypoxia produced sex-dependent phenotypes of inflammation and oxidative stress, likely dependent on mitochondrial oxidative stress [55]. Similarly, intrauterine exposure to hypoxia induced long-term alterations in cardiac mitochondrial respiration specifically in female offspring compared to males [56].

In addition, only five out of the thirteen subjects enrolled in our study had spO_2_ ≤ 80% at t-hypoxia. As previously suggested, this result is partly due to our hypoxia protocol, in which the subjects were exclusively at rest. Based on our findings, it would be interesting to perform another study with a larger sample size and to expand the hypoxia protocol with both rest and exercise conditions.

In addition, as our analysis involved healthy subjects with no recent or current infections and no chronic diseases, we did not perform a blood cell count. However, given the half-life of PBMCs, we can assume that the number of blood cells was stable during the different samples at t-start, t-hypoxia and t-60′ [57]. Finally, as PBMCs comprise different cell types, it would be interesting in the future to correlate complex II mitochondrial respiration with the proportion of monocytes or lymphocytes, which play different roles in inflammation and might respond differently to hypoxia [49].

## 4. Material and Methods

### 4.1. Population and Methods

This study prospectively enrolled 13 healthy volunteers, non-smokers or ex-smokers, aged from 18 to 50, at the Physiology and Functional Exploration Unit, University Hospital of Strasbourg, France. All subjects had no current or recent infection and a normal physical examination, electrocardiogram and hemoglobin and were not receiving any medical treatment. All subjects gave written consent before participation. The study was conducted in accordance with the tenets of the Declaration of Helsinki and was approved by the National Ethic Committee (CPP Ile de France 11, 21-048-34245 received 1 August 2021) and registered on ClinicalTrials.gov: NCT05044585.

### 4.2. Study Design

**Figure 6:**
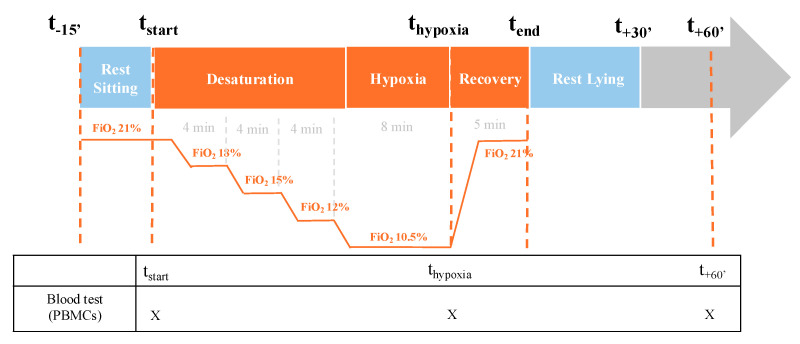
Study design. FiO_2_: fraction of inspired oxygen; min: minutes; PBMCs: peripheral blood mononuclear cells.

The sequence of the hypoxia test is summarized in Figure 6. After a 15 min control period at rest (t-15′), the hypoxia program was initiated by gradually decreasing ambient air (FiO_2_ 21%) to 10.5% FiO_2_ using a controlled gas flow (AltiTrainer Isocap device, Sport et Medical technologies SA, GANSHORN Schiller Group, Germany). The FiO_2_ was thus decreased by 3% steps, with a plateau held for 4 min at each step, starting from a stabilized FiO_2_ of 21% (before desaturation was started) to 18% (first plateau), 15% (second plateau) and 12% (third plateau). The final plateau lasted 8 min at 10.5% FiO_2_. After this step, the subjects were given ambient air (FiO_2_ of 21%) to return to baseline.

To enable blood analysis (mitochondrial respiration and H_2_O_2_ production by PBMCs), a peripheral catheter was inserted into a brachial vein at baseline, before hypoxia. Three blood samples were collected at defined intervals: baseline t-start (before the start of the test), t-hypoxia (at the end of the last plateau of hypoxia), t + 60′ (60 min after the return of normal air, when the subjects had reached their initial normal saturation).

### 4.3. Determined Parameters

#### 4.3.1. Clinical Parameters

The participant’s physiological parameters monitored during the test included the following:

Oxygen levels, monitored continuously with oxygen saturation measured by non-invasive pulse oximetry (SpO_2_);

Heart rate;

Respiratory rate by airflow analysis;

Blood pressure (in mmHg) using an adjusted upper arm cuff.

#### 4.3.2. Mitochondrial Respiration of PBMCs

PBMCs were extracted by the Ficoll method, as previously described [15]. Oxygen consumption of 2.5 × 10^6^ PBMCs/mL was determined using a high-resolution oxygraph (Oxygraph-2k; Oroboros Instruments, Innsbruck, Austria) at 37 °C with continuous stirring. PBMCs’ mitochondrial complex respiration was determined according to the following protocols, in which different substrates and inhibitors were successively introduced into the chamber of the oxygraph:

Protocol 1 (analysis of global mitochondrial respiration) determined (1) the basal oxygen consumption ratio (OCR) by exposing the cells to glutamate (5 mM) and malate (2 mM), (2) the maximum uncoupled OCR by the addition of the uncoupler carbonyl cyanide-p-trifluoromethoxyphenylhydrazone (FCCP 0.25 µM), and (3) the RCR as an index of the coupling of ETC respiration to ATP production. These were determined by dividing the oxygen uptake rate in state 3 (respiration at maximum uncoupled OCR with FCCP minus non-mitochondrial OCR measured by adding the electron transport inhibitors rotenone (0.5 µM) for complex I and antimycin A (2.5 µM) for complex III) by state 4 (OCR due to proton leak measured by adding the ATP synthase inhibitor oligomycin (2.5 µM)).

Protocol 2 was as follows: (1) First, the cell membranes were permeabilized with saponin (125 µg/mL) and complex I was activated with glutamate (5 mM) and malate (2 mM). ADP (2 mM) was then added to study OXPHOS via the electron transport coming from complex I. (2) Succinate (25 mM), an activator of mitochondrial complex II, was then added to study OXPHOS via complexes I and II (CI + II OXPHOS). (3) Rotenone (0.5 μM), an inhibitor of complex I, enabled the analysis of CII OXPHOS. (4) Finally, N,N,N′,N′-Tetramethyl-p-phenylenediamine dihydrochloride (TMPD)/ascorbate (0.5 mM/0.5 mM) was added to provide electrons to complex IV, enabling the preferential study of the CIV OXPHOS.

All O_2_ consumption results are expressed in pmol/s/10^6^ cell.

#### 4.3.3. Measurement of Mitochondrial H_2_O_2_ Production

To investigate ROS production in PBMCs, we measured H_2_O_2_ production during ETC respiration (CI, CI + II and CII OXPHOS), using the fluorescent probe Amplex Red (20 µM) and Horseradish Peroxidase (1 U/mL). As TMPD/ascorbate are strongly redox-active substances, H_2_O_2_ was not analyzable for CIV OXPHOS [58].

### 4.4. RT-PCR

Quantitative PCR was performed using the QuantStudio 3 Real-Time PCR System (Applied Biosystem, Thermo Fisher Scientific, Waltham, MA, USA) and PowerTrack SYBR Green Master Mix (Applied Biosystem). Beta-2-microglobulin was used as an internal control.

Five genes were analyzed: HIF-1α, STAT3, CXCL9, ISG15, and SUCNR1, Table 2.

### 4.5. Statistical Analysis

Statistical analyses were performed using Prism 9 software (GraphPad). Continuous variables are expressed as mean ± standard error of the mean (SEM). As each subject was its own control, comparisons were made using the non-parametric Friedmann test for repeated measures. Correlations were analyzed using a non-parametric Spearman’s test. A *p*-value < 0.05 was considered significant.

## 5. Conclusions

In summary, these original data support that acute severe hypoxia impairs the mitochondrial respiratory chain function, by rapidly decreasing complex II activity in PBMCs. Further, the association between reduced mitochondrial complex II respiration and the succinate–HIF axis and inflammation improves our understanding of hypoxia pathophysiology in humans.

Since hypoxia and inflammation are also hallmarks of many diseases including myocardial injury and more generally cardiovascular diseases, further studies will be useful to investigate whether modulation of complex II activity may modulate inflammatory and metabolic alterations observed during heart failure.

## Figures and Tables

**Table 1:** Cardiorespiratory effects of hypoxia.

	Normoxia (FiO_2_ 21%)	Effect of Hypoxia (FiO_2_ 10.5%)		*p*
	N = 13 Total Population	N = 8 with spO_2_ > 80%	N = 5 with spO_2_ ≤ 80%	
spO_2,_ %	99 ± 1	83.5 ± 1.4	76.8 ± 1.1 ^$^	* <0.0001
Respiratory rate, /min	13 ± 0.8	12 ± 1	11 ± 1	* 0.06
Minute ventilation, L/min	10.2 ± 0.7	12.8 ± 1.1	9.2 ± 1.5 ^$^	* 0.30
Heart rate, /min	73 ± 2	81 ± 3	84 ± 6	* 0.04
Respiratory rate variation between t0 and t-hypoxia		−2.5 ± 1	−2.5 ± 0.5 ^$^	^$^ >0.99
Minute ventilation variation between t0 and t-hypoxia		+2.5 ± 1.3	−0.7 ± 0.8 ^$^	^$^ 0.14

FiO_2_: fraction of inspired oxygen; spO_2_: oxygen saturation measured by non-invasive pulse oximetry. * Comparison between total population values in rest normoxia and rest hypoxia ^$^ Comparison of values between the 2 groups of subjects (with spO_2_ ≤ or > 80%). For spO_2_ and minute ventilation, *p* = 0.001 and *p* = 0.06, respectively. All the results are expressed as mean ± SEM.

**Table 2:** Primer sequences of the genes analyzed in RT-PCR.

Gene	Forward	Reverse
CXCL9	TAAGCGCTAGAGGAAGCAGC	TTCACTGAACCTCCCCTGGA
HIF-1α	CCAGACGATCATGCAGCTACT	TGATTGCCCCAGCAGTCTAC
ISG15	GATCACCCAGAAGATCGGCG	GGATGCTCAGAGGTTCGTCG
STAT3	GGAACAAGCCCCAACCGGA	CTAAAATCAGGGGTCCCAACTGT
SUCNR1	CGTTGTGGGAGTCCTTGGAA	GGTTGCTTATGCAGAGCACG

## Data Availability

M.R. and B.G. are the guarantors of the content of the manuscript, including the data and analysis.
